# The Sharing Experimental Animal Resources, Coordinating Holdings (SEARCH) Framework: Encouraging Reduction, Replacement, and Refinement in Animal Research

**DOI:** 10.1371/journal.pbio.2000719

**Published:** 2017-01-12

**Authors:** Bethny Morrissey, Karen Blyth, Phil Carter, Claude Chelala, Louise Jones, Ingunn Holen, Valerie Speirs

**Affiliations:** 1 Leeds Institute of Cancer and Pathology, University of Leeds, Leeds, United Kingdom; 2 Cancer Research UK Beatson Institute, Glasgow, United Kingdom; 3 Barts Cancer Institute, London, United Kingdom; 4 Academic Unit of Clinical Oncology, University of Sheffield, Sheffield, United Kingdom

## Abstract

While significant medical breakthroughs have been achieved through using animal models, our experience shows that often there is surplus material remaining that is frequently never revisited but could be put to good use by other scientists. Recognising that most scientists are willing to share this material on a collaborative basis, it makes economic, ethical, and academic sense to explore the option to utilise this precious resource before generating new/additional animal models and associated samples. To bring together those requiring animal tissue and those holding this type of archival material, we have devised a framework called Sharing Experimental Animal Resources, Coordinating Holdings (SEARCH) with the aim of making remaining material derived from animal studies in biomedical research more visible and accessible to the scientific community. We encourage journals, funding bodies, and scientists to unite in promoting a new way of approaching animal research by adopting the SEARCH framework.

## Introduction

The contributions that animal models have made to biomedical research and their translation to human health and welfare are undisputed. Many significant medical milestones, including the development of antibiotics (e.g., penicillin [1]), vaccines (e.g., the polio vaccine [2]), medications for chronic diseases (e.g., insulin for diabetes [3]), surgical techniques (e.g., blood transfusions and organ transplants [4]), and the development of breast cancer therapies such as tamoxifen and trastuzumab (Herceptin) [5–7], have all been achieved because of breakthroughs made possible through using animals in research. However, these advances often come at a high cost to biomedical researchers both financially and in time spent in development. These research costs are increasing at a time when financial resources that governments, charities, and other funding organisations have available to support research remain static [8]. As it is unlikely that these financial restrictions will lift any time soon, researchers are encouraged to start thinking laterally about how they can increase the quality, depth, and impact of their research in a low-cost, sustainable way. In addition to this, in most developed countries, researchers who utilise animals in research are legally obliged to consider what are known as the 3Rs in animal research: Replacement, Reduction and Refinement.

### Current Trends in Animal Use in United Kingdom Biomedical Research

**Fig 1:**
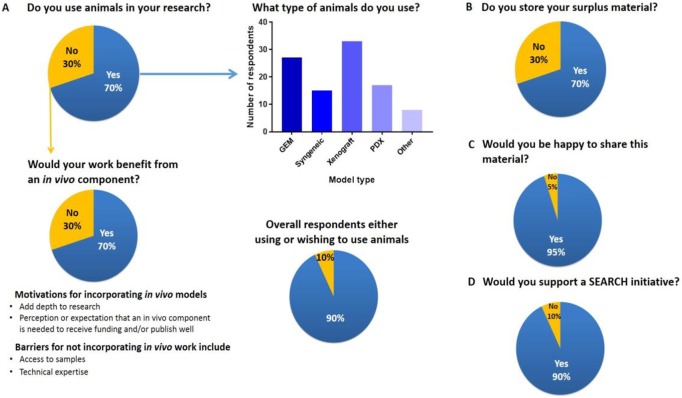
Summary of responses to our Bristol Online Survey. Participants were asked (A) if they used animals in research, their type (GEM, genetically engineered mouse; PDX, patient-derived xenograft), and if their work would benefit from an in vivo component; (B) if they stored surplus material; (C) if they would be willing to share this material; and (D) if they would support a SEARCH initiative.

In order to gauge trends in the use of animals in biomedical research, we devised a Bristol Online Survey (BOS), which was distributed electronically to over 100 biomedical researchers representing more than 90 research institutes or groups. The questions posed aimed to establish how frequently animals were used in oncology, neuroscience, cardiology, and other disciplines and, if not used, if there was a benefit in pursuing this and the motivation behind that choice. Key questions and responses are illustrated in Fig 1, with all ten questions posed listed in S1 Appendix.

Of 135 respondents, 70% currently use animals in their research, and of those who do not, 70% wished to; thus, 90% of the total population surveyed would benefit from using animals in their research. Our survey therefore suggests that the number of animals used in research could rise over the coming years. This reflects the findings from the European Commission that the net number of animals used across Europe in cardiovascular and oncology research rose to 115,000 and 250,000, respectively, between 2008 and 2011 [9]. From our survey, the main motivations behind wanting to incorporate animal models included a perceived benefit to the depth and quality of the research. However, there was also a perception or expectation that an in vivo component is needed to obtain funding and to publish in high-impact journals. This predicted rise in the use of animals in research is likely to be of concern to groups and government organisations focussed on implementing the 3Rs.

For survey respondents who do not use animals currently but wished to do so, the main reasons listed included lack of access to tissue and/or technical expertise and financial constraints. As most of those using animal models had surplus archival material, this led us to consider if it might be possible to find a way to facilitate the removal of these barriers without the need to use more animals.

### Developing the SEARCH Framework

Clearly many researchers are already addressing the 3Rs; however, concerns have been raised in the community about their appropriate implementation. For example, in some instances, the number of animals has been reduced to such an extent that there are concerns this may be compromising the robustness of experimental data being generated [10].

Our BOS highlighted that there is significant surplus material generated from most animal studies. This is often archived in-house, stored indefinitely, and frequently never revisited, hence representing a considerable untapped resource; in our survey, two-thirds of respondents currently using animals in their research reported storing excess tissue upon study completion. Encouragingly, 95% of the researchers surveyed were willing to share this material on a collaborative basis. It would make economic, ethical, and academic sense to explore the option to utilise this precious resource in the first instance, before generating new/additional animal models and associated samples, if they already exist elsewhere [11].

To bring together those requiring animal tissue and those holding this type of archival material, we have devised a framework called Sharing Experimental Animal Resources, Coordinating Holdings (SEARCH) with the aim of making leftover material derived from animal studies in biomedical research more visible and accessible to the scientific community [12].

**Fig 2:**
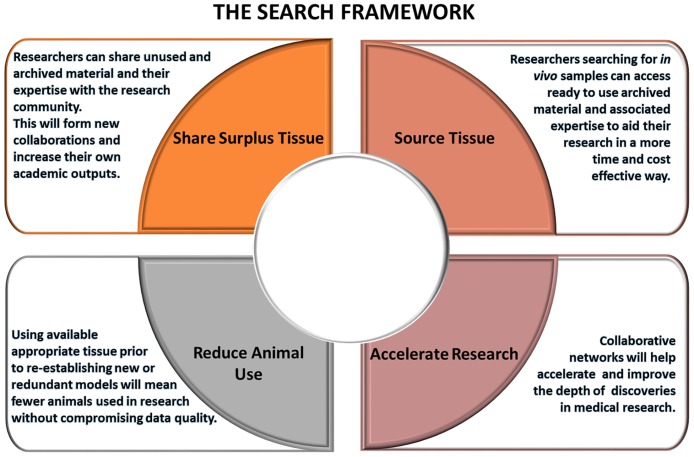
Diagram of the SEARCH framework to connect researchers, share materials, and accelerate discovery whilst reducing animal use.

The SEARCH framework contains four policies, described below and illustrated in Fig 2.

#### 1. Share surplus tissue

Crucial to the success of the SEARCH framework is willingness of researchers to share their archived reserves with the research community. Sharing resources has the potential to foster new collaborations, which may not be immediately apparent through current networks, hence increasing research impact. These benefits come at minimum cost to the group requiring the material and mean that the investment by the person who has developed the material has added value.

#### 2. Source tissue

SEARCH facilitates a web-based database through which scientists can search and identify models that best fit their planned research. This echoes Shared Ageing Research Models (ShARM; http://www.ShARMUK.org), an online resource that facilitates access to aged murine models and their tissues. However, unlike SEARCH, ShARMUK collects, stores, and distributes tissues from aged murine models through its biorepository and maintains a database of live ageing mouse colonies. With SEARCH, the burden of maintaining expensive colonies and storing materials, plus their associated costs, is relieved. SEARCH acts as a free, no-cost partnership broker for those looking for material by connecting them to those who have suitable archived samples. Any associated material transfer agreements and other considerations (e.g., how the tissues may have been fixed or stored and authorship inclusion agreements) are up to the respective researchers and their institutions. SEARCH will enable those who lack in vivo expertise or infrastructure the ability to perform experiments without the need to start from first principles, saving considerable time and resources.

#### 3. Accelerate research

An important part of the SEARCH ethos is the generation of networks of researchers who are willing to connect and share their expertise and resources. SEARCH promotes collaboration over competition, and by doing so, we believe scientific discoveries will be accelerated. Furthermore, in a funding-restricted climate, the ability to bypass the high cost of generating complex animal models will save researchers time and money. These collaborations are also enabling researchers to complete pilot data studies in a short space of time with minimal resources that are being used to strengthen grant applications, aiding early-career researchers in accelerating their careers.

#### 4. Reduce animal use

At the crux of the SEARCH initiative is the belief that high-quality research can still be achieved whilst reducing the number of animals used in biomedical research. This can be achieved by preferentially using suitable archived material, without the need to generate unnecessary new models, also helping to maintain the robustness of results whilst using fewer animals.

### SEARCHBreast: The First SEARCH Prototype

The SEARCH initiative requires two crucial pieces of infrastructure: a secure database whereby researchers can enter information about material available to share and an accompanying website to host the database. We have developed this for our prototype, SEARCHBreast (https://searchbreast.org/; https://www.youtube.com/watch?v=F_1mDBjEHe4), launched in 2014. This has resulted in sign-ups from over 220 scientists to date, from the UK, European Union, United States, and Australia, who all support the ethos of sharing resources and expertise to accelerate breast cancer research [12].

The SEARCHBreast portfolio contains tissues from xenograft and genetically engineered models and also includes an extensive range of patient-derived xenograft (PDX) models. In light of the recent decision by the US National Cancer Institute (NCI) to replace their NCI-60 cell line screen with PDX models [13], it is pertinent that breast PDX models are made available to the community, particularly as these can be difficult to produce, requiring direct access to human clinical material to establish, and come at a high cost to generate and maintain over a long period of time. To our knowledge, SEARCHBreast is currently one of the only freely accessible resources from which PDX models are available to share on a no-cost, collaborative basis, which is clearly advantageous to the breast cancer community. As of 31 October 2016, 86 animal models were available through SEARCHBreast, representing thousands of ready-to-use tissue samples, including formalin-fixed paraffin-embedded tissue blocks, cells, and histological slides [12].

### The Potential of the SEARCH Framework

**Fig 3:**
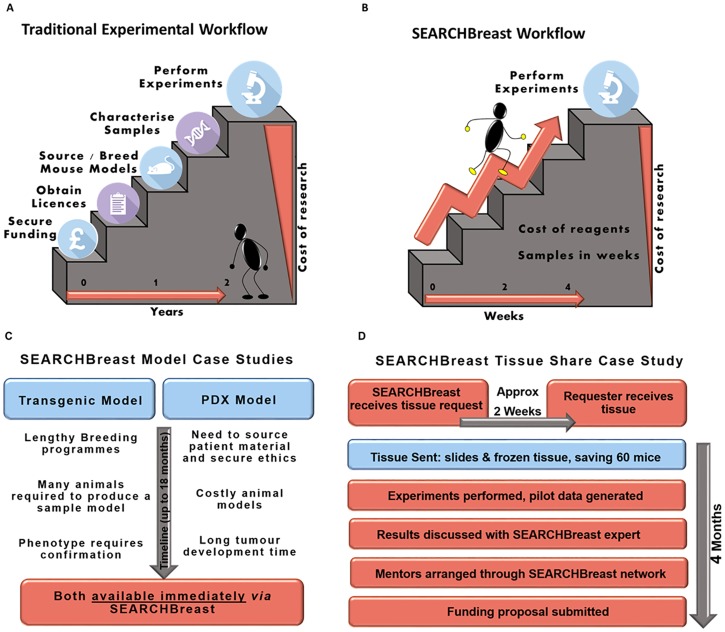
SEARCH discovery pipeline. (A) Traditional experimental workflow for in vivo research. (B) Advantages of utilising the faster and more streamlined SEARCH workflow. (C) Case study 1 showing the advantages of using models deposited in SEARCHBreast. (D) Case study 2 with an example of how SEARCH can accelerate tissue sharing and experimental discovery.

Using SEARCHBreast as an example, the benefits of using the SEARCH framework are shown in Fig 3. For example, the Blg-*Cre*:*Brca1*^f/f^:Tr*p53*^+/-^ genetic model of breast cancer (available on the SEARCHBreast database) would require an extensive breeding programme just to generate a single mouse with the required phenotype, which may take up to 18 months to manifest and be of considerable expense [14]. By contrast, accessing this tissue from SEARCHBreast reduces this to a matter of weeks, with no further animals required, at a considerably lower cost and requiring only those reagents needed for the experiments.

Since its inception in 2014, SEARCHBreast has facilitated the sharing of hundreds of tissue samples from a range of different types of models. This has resulted in sparing 400 animals, including those which would have been culled during transgenic breeding programmes. We predict this number will rise significantly with increasing awareness and adoption of the resource by the scientific community. Consequently, not only does SEARCHBreast have the capacity to save researchers time and money whilst increasing the potential impact of their research, but it addresses the reduction arm of the 3Rs. Similarly, it also aims to address replacement and refinement. The SEARCHBreast website contains links to resources, designed to ensure that researchers are well versed on the latest refinement measures in animal studies. Additionally, SEARCHBreast is committed to promoting the uptake of emerging technologies, e.g., through 3D tissue modelling [15], which may reduce, or even replace, the need for animals in research and instead promote a focus on using human tissues and cells available via biorepositories such as the Breast Cancer Now Tissue Bank cell culture programme (http://www.breastcancertissuebank.org/) or the Komen Tissue Bank (http://komentissuebank.iu.edu/). Indeed, the SEARCHBreast team held a workshop on 3D breast cancer modelling in May 2015 to promote this type of work, with many of the presentations available on the website (https://searchbreast.org/) and the workshop proceedings published as well [15].

## Summary

With a perception that animal models are required to generate high-impact publications, a mind-set that will be challenging to reverse and which is beyond the scope of this article, the SEARCH framework offers a fresh way of thinking about in vivo research. As exemplified by those who have signed up to SEARCHBreast and in our BOS that addressed wider disease types, there is support by the scientific community for the complementary approach to animal research we are proposing. Furthermore, the platform we have developed for SEARCHBreast is sufficiently generic to allow easy expansion into other disease types.

Despite recognised benefits in sharing resources, there is often some reluctance by scientists to adopt this fully [16]. We believe that engagement with the breast cancer community from the project’s inception was crucial to our success in establishing SEARCHBreast. This was an iterative process to ensure we developed a resource that was fit for purpose for the research community and would be embraced by them. Furthermore, by making the resource open to all—researchers do not need to deposit a model to be able to use SEARCHBreast—we have promoted inclusivity. Further still, both parties can benefit: the depositor sees his/her model(s) put to additional use and may gain added value in terms of publications, while the user has access to materials that would otherwise be costly and time consuming for him/her to develop from scratch, thus overcoming some of the practical barriers that may be associated with resource sharing.

On the back of the success of SEARCHBreast and akin to the ARRIVE guidelines, which encourage comprehensive reporting on experiments and outcomes of research in animals [17], we would like to recommend that funding bodies support building the SEARCH framework to encourage appropriate cataloguing and storing of surplus materials, at the same time encouraging researchers to log their surplus materials at the conclusion of animal studies to make this visible and accessible to others. While data sharing plans are frequently requested at the time of submitting a funding application, details on what happens to surplus animal materials and how these may serve the wider research community at the conclusion of a project tend to be overlooked, despite recommendation from experts to do so [18]. As such we present a checklist that funding bodies might consider to assist in the grant evaluation process (S2 Appendix). We invite the *PLOS Biology* readership to adopt the SEARCH blueprint and unite in sharing animal tissues in a more effective way.

## Supporting Information

S1 Appendix Proposed checklist for funding bodies to consider for research involving animal models. (DOCX)

S2 Appendix Questions posed in the Bristol Online Survey. (DOCX)

## Abbreviations

3Rs: Replacement, Reduction and Refinement
BOS: Bristol Online Survey
GEM: genetically engineered mouse
NCI: National Cancer Institute
PDX: patient-derived xenograft
SEARCH: Sharing Experimental Animal Resources, Coordinating Holdings
ShARM: Shared Ageing Research Models
