# Supplementary material for: The Sharing Experimental Animal Resources, Coordinating Holdings (SEARCH) Framework: Encouraging Reduction, Replacement, and Refinement in Animal Research
Source: PLoS Biol. 2017 Jan 12;15(1):e2000719. doi: 10.1371/journal.pbio.2000719 (PMC5230739; doi:10.1371/journal.pbio.2000719)
Supplement: S1 Appendix — (DOCX) [file pbio.2000719.s001.docx]

**S1 Appendix**

**Questions posed in the Bristol Online Survey**

1. **What is your research area?**

Cancer research

Cardiovascular disease

Reproductive research

Pulmonary disease

Neurological disease

Diabetes and endocrinology

Other (please specify)

1. **Do you use animals in your research?**

Yes

No

1. **What type of animals do you use?**

Genetically modified mouse models

Syngeneic mouse models

Xenograft mouse models

Patient-derived xenograft (PDX) models

Other (please specify)

1. **From your *in vivo* work do you have surplus archived animal materials which is stored in some way e.g. frozen, formalin-fixed paraffin-embedded, PDX, cell culture etc.?**

Yes

No

1. **Would you be willing to share this stored material with other researchers on a collaborative basis?**

Yes

No

1. **What are the reasons for not storing or archiving animal material from your research?**

No viable material left after experiments

No facilities for storage

Other

1. **What are the reasons preventing you from sharing material?**

Study still in progress/work unpublished

May still need the materials in future

Wish to protect intellectual property

Other

1. **Would your research benefit from an *in vivo* component?**

Yes

No

1. **What are the barriers in incorporating *in vivo* work into your research?**

Access to samples

Lack of technical expertise

Don’t know where to start

Other

1. **Would you support a virtual on-line bioresource designed to facilitate the sharing of surplus archived animal material across various human diseases?**

Yes

No
