# Supplementary material for: The Sharing Experimental Animal Resources, Coordinating Holdings (SEARCH) Framework: Encouraging Reduction, Replacement, and Refinement in Animal Research
Source: PLoS Biol. 2017 Jan 12;15(1):e2000719. doi: 10.1371/journal.pbio.2000719 (PMC5230739; doi:10.1371/journal.pbio.2000719)
Supplement: S2 Appendix — (DOCX) [file pbio.2000719.s002.docx]

**S2 Appendix**

**Proposed checklist for funding bodies to consider for research involving animal models**

| Do you intend to store all of the tissues which may remain at the conclusion of your research or only a proportion e.g. sufficient for repeat assays should reviewers ask for this? |
| --- |
| Is there appropriate physical space to store these tissues? |
| Is there a cost to store these tissues and if so has this been included in your budget? |
| Are samples labelled appropriately to allow identification and linkage to original experiments? |
| Is there a secure, searchable database for sample cataloguing to allow identification and retrieval? |
| Do you have the capacity (staff) to retrieve and ship samples if collaborators request to share material? |
| What happens to these tissues if you leave the institution? |
